# Supplementary material for: Microwave-Solvothermal Synthesis of Mesoporous CeO2/CNCs Nanocomposite for Enhanced Room Temperature NO2 Detection
Source: Nanomaterials (Basel). 2024 May 7;14(10):812. doi: 10.3390/nano14100812 (PMC11124451; doi:10.3390/nano14100812)
Supplement: Supplementary file 1 [file nanomaterials-14-00812-s001.zip › nanomaterials-2981728-supplementary.pdf]

Supporting Information

Microwave-solvothermal synthesis of mesoporous CeO_2/CNCs nanocomposite for room temperature NO_2 detection

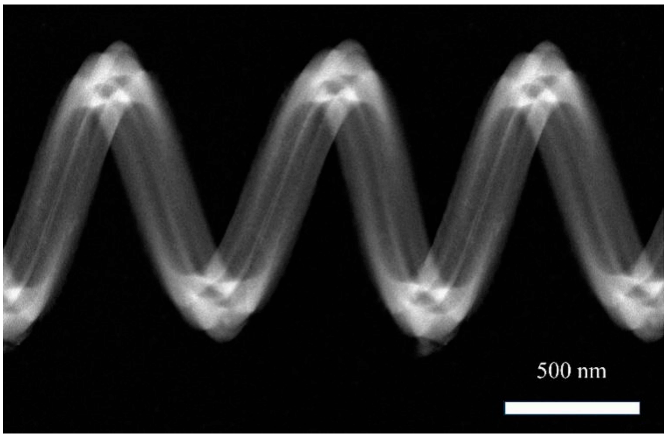

Figure S1. TEM image of CNCs

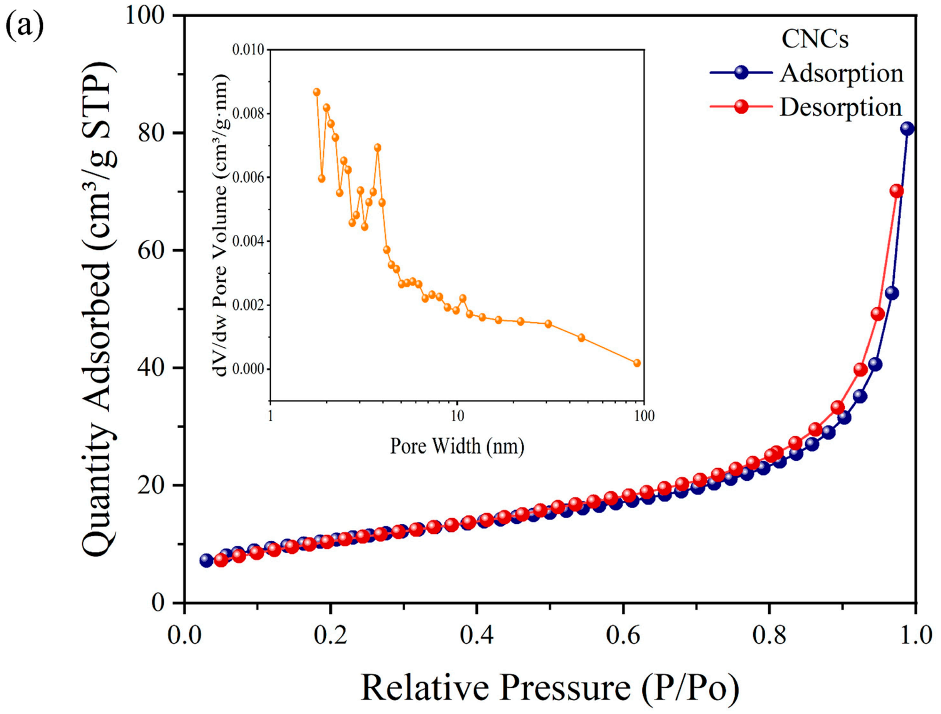

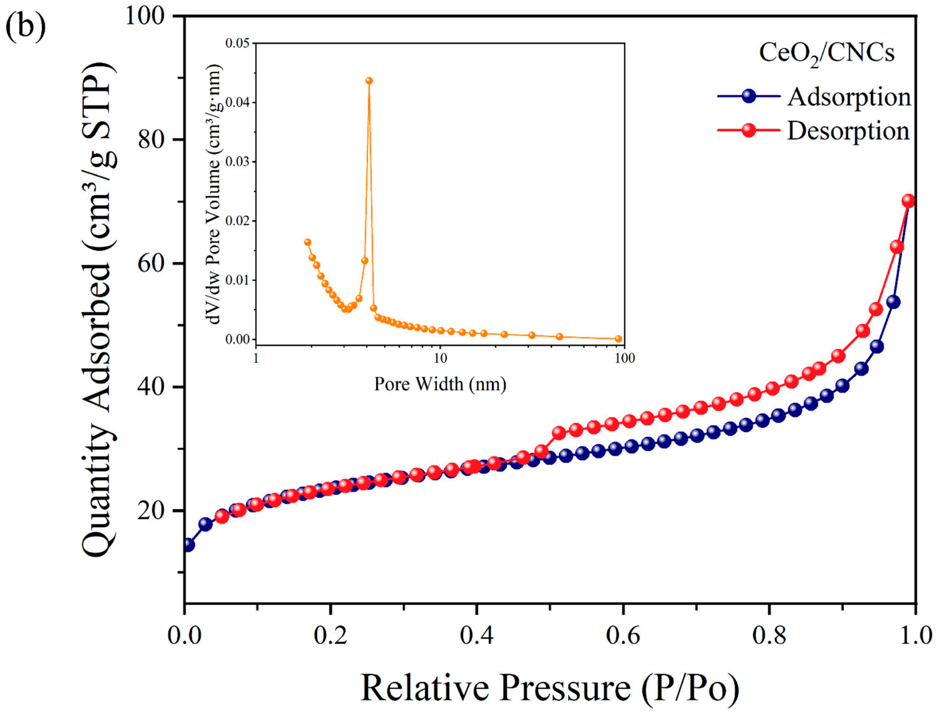

Figure S2. The N_2 adsorption and desorption isotherms and pore size distribution (the inset) curve of bare CNCs (a) and (b) CeO_2/CNCs nanocomposite

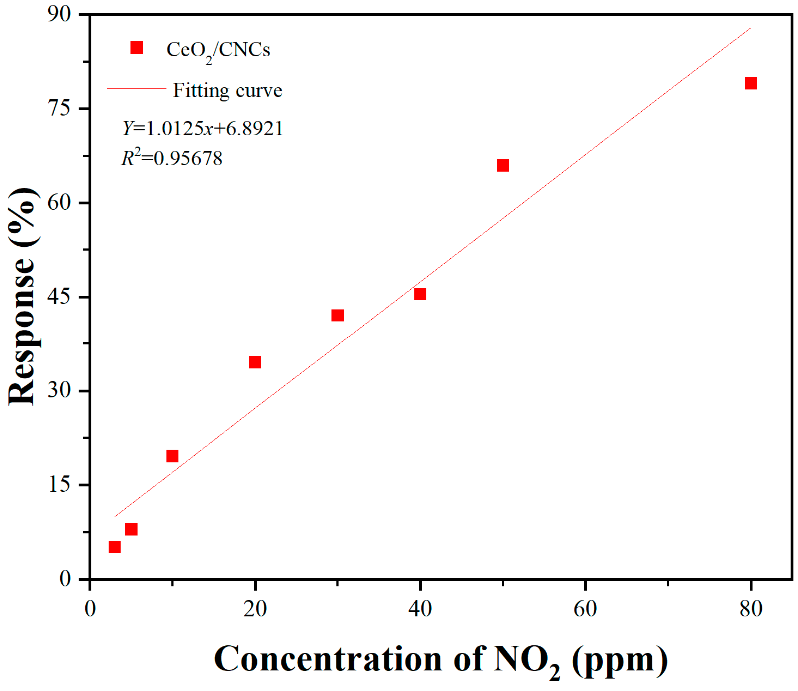

Figure S3. Fitting curve of the CeO_2/CNCs composites

The process of calculation of noise level (RMS_{Noise}) and limit of detection (LOD):[S1]

$$RMS_{Noise}\left(ppm^{-1}\right)=\sqrt{V_x^2/n} \quad \text{where} \quad V_x^2=\sum\left(Y_i-\overline{Y}\right)^2 \tag{1}$$

$$Limit\ of\ detection\,(ppm)=3\times\frac{RMS_{Noise}}{Slope} \tag{2}$$

[S1] Duy, L. T.; Kim, D. J.; Trung, T. Q.; Dang, V. Q.; Kim, B. Y.; Moon, H. K.; Lee, N. E. High Performance Three-Dimensional Chemical Sensor Platform Using Reduced Graphene Oxide Formed on High Aspect-Ratio Micro-Pillars. Adv. Funct. Mater. 2015, 25, 883–890.

Table S1. The ratios of cerium and oxygen of as-prepared samples.

Sample	O_{lat}	O_{vac}	O_{ads}	Ce^{3+}	Ce^{4+}
Sample 1	84.6	14.11	1.29	47.98	52.02
Sample 2	65.25	29.52	5.23	37.36	62.64
Sample 3	56.49	35.6	7.91	22.42	77.58
Sample 4	53.02	34.69	12.29	19.38	80.62
